# Improving reproducibility in computational biology research

**DOI:** 10.1371/journal.pcbi.1007881

**Published:** 2020-05-19

**Authors:** Jason A. Papin, Feilim Mac Gabhann, Herbert M. Sauro, David Nickerson, Anand Rampadarath

**Affiliations:** 1 Department of Biomedical Engineering, University of Virginia, Charlottesville, Virginia, United States of America; 2 Department of Biomedical Engineering, Johns Hopkins University, Baltimore, Maryland, United States of America; 3 Department of Bioengineering, University of Washington, Seattle, Washington, United States of America; 4 Auckland Bioengineering Institute, University of Auckland, Auckland, New Zealand

There has been much discussion in the scientific literature on a crisis of reproducibility in science [1, 2]. It has been reported that the percentage of studies that are reproducible is as low as 10% or less, depending on the discipline [3]. This inability to reproduce scientific findings from a given paper has been attributed to a lack of clarity in the methods and inherent variability in the biological system being studied [4].

Reproducibility in computational biology research is certainly a problem, yet perhaps a challenge that our field can uniquely tackle. A lack of reproducibility in computational biology research can be attributed to many factors, but incomplete or erroneous descriptions of the simulations (e.g., which software version was used), incomplete documentation on how to run simulations, or simply failing to post the relevant computer code needed to run a given simulation are common issues that occur.

Many tools have emerged that we can leverage to make computational biology research more reproducible (e.g., http://co.mbine.org/ and https://normsys.h-its.org/) and there exist articles that propose best practices, such as Ten Simple Rules for Reproducible Computational Research [5] or Ten Simple Rules for Writing and Sharing Computational Analyses in Jupyter Notebooks [6].

*PLOS Computational Biology* recently partnered with the Center for Reproducible Biomedical Modeling (https://reproduciblebiomodels.org/) to launch a pilot peer review workflow to assess reproducibility (https://blogs.plos.org/biologue/2020/05/05/improving-reproducibility-of-computational-models/). After authors opt-in to participation in the pilot, a peer reviewer will be solicited (in addition to our normal peer review assessment) to specifically evaluate the reproducibility of the computational modeling aspects described in the submission. All the peer reviewers can receive credit for the reviews through our partnership with ORCID (https://blogs.plos.org/plos/2019/06/youve-completed-your-review-now-get-credit-with-orcid/) and authors can elect to have the peer reviews published alongside the final publication of the work (https://blogs.plos.org/plos/2019/05/plos-journals-now-open-for-published-peer-review/). We aim to have the review process completed in the usual time frame with a hope that the additional review will help editors and authors assess and improve the reproducibility of the work. There are a few questions we intend to investigate with this pilot, including questions the pilot can answer directly:

- How popular is the desire to publish reproducible models?
- What is the minimum information required to reproduce published model results?
- What are the common reasons computational studies are not reproducible?

and questions that the pilot will help us start to explore and point us in the right direction:

- What are the benefits of producing reproducible models?
- What incentives would attract authors to make the studies available in a reproducible manner?
- What tools and technologies can be created to facilitate reproducibility?
- What training is required to improve community awareness of tools and practices which lead to FAIR (Findable, Accessible, Interoperable, Reusable) computational studies?

We feel the answers to these questions can help inform the community how best to encourage a change in culture toward a more FAIR [7] computational biology.

There are already some general principles we’ve learned about good practices for reproducible biomedical modeling [8]. These include stating the software used in the study, including the particular version used, providing machine readable code in supplements or uploaded to established repositories, and asking a third party to test that your methods section is free from error and of sufficient detail to reproduce the results presented in the paper.

We are certain to learn more as this pilot progresses. We will need to tackle challenges like developing and supporting repositories for the models, scaling up the “reproducibility validation” peer review effort, and creating tools to help make these assessments. As a community, we need to learn how to properly recognize the tremendous effort of reviewers involved in this work, perhaps with increased support of ORCID or Publons as tools to help peer reviewers receive credit for their work in the assessment and publication process. We need to work out how papers that have been so vetted are appropriately identified, perhaps with the use of badges that provide a “stamp of approval” for papers and associated codes that have been assessed and passed defined criteria to provide public recognition.

We think that journals and scientific publications have a critical part to play in making scientific work more reproducible. As a journal for the computational biology research community, PLOS Computational Biology is working to address this significant need within the community. As our work is more rigorously evaluated for reproducibility, we can build on each other’s contributions to advance science.
